# Sarcoidosis and autoimmunity: In the depth of a complex relationship

**DOI:** 10.3389/fmed.2022.991394

**Published:** 2022-09-06

**Authors:** Luigi Rizzi, Carlo Sabbà, Patrizia Suppressa

**Affiliations:** Department of Internal Medicine and Rare Diseases Centre “C. Frugoni”, University Hospital of Bari, Bari, Italy

**Keywords:** sarcoidosis, autoimmunity, vimentin, proteomics, rare disease

## Abstract

Sarcoidosis is a chronic granulomatous disease that can virtually affect any organ. Its etiology is unknown, although it has been proposed that environmental or biological agents can act as triggers, ultimately leading to chronic inflammation in genetically predisposed individuals. The main component of sarcoid inflammation is represented by an exaggerated T- lymphocytic cellular response to a putative antigen that could not be efficiently cleared in the patient. However, several clinical and immunological observations, such as the association of sarcoidosis to autoimmune diseases or the presence of autoantibodies in the serum of patients with sarcoidosis, suggest that humoral-mediated immune response might also play a role in the pathogenesis of sarcoidosis. The aim of this review is to deepen the relationship between sarcoidosis and autoimmunity, by analyzing the most recent advances and proposing new fields of research.

## Introduction

Sarcoidosis is an inflammatory disease with variable clinical course, characterized by the presence of non-necrotizing granulomas in the involved organs; virtually, any organ can be affected, although the lung is one of the most frequently involved sites. The incidence of the disease varies widely depending on geographical area, showing the highest rates among Scandinavians and African Americans and the lowest ones in Asian countries; the average age of onset typically lies between 40 and 55 years of age. Etiology is unknown, even though it has been hypothesized that microorganisms, environmental factors, and inorganic materials could act as trigger(s) for inflammation in genetically predisposed individuals (1).

The clinical course of sarcoidosis includes acute, chronic but stable, or chronic and progressive disease. In half of the cases, the disease resolves spontaneously within 2 years; in many other patients, remission occurs within 5 years, whereas a remission after 5 years is unlikely (2).

Clinical presentation is widely heterogeneous: symptoms are generally non-specific, including cough, chest discomfort, dyspnea, low-grade fever, tiredness, weight reduction, and night sweats. On the other hand, sarcoidosis can be often entirely asymptomatic or, conversely, determines specific symptoms in relation to the involved organs. In particular, lung involvement is observed in more than 90% of patients, generally with hilar or mediastinal lymph nodes enlargement and parenchymal infiltration. Skin involvement occurs in 20–30% of patients: erythema nodosum, profuse sweating, nodules, papules, and plaques are the most frequent manifestations (3). Regarding eye involvement (10–50% of patients), uveitis is the commonest form of ocular manifestation. Cardiac, neurological, renal, musculoskeletal, or gastrointestinal involvements are also possible (4).

Sarcoidosis symptoms can also be framed in two syndromic diseases: Löfgren syndrome, highly suggestive for sarcoidosis, which is characterized by fever, bilateral ankle arthritis, and/or erythema nodosum and bilateral hilar lymphadenopathy diagnosed using chest radiography (5); Heerfordt syndrome is characterized by uveitis, enlargement of the parotid and submaxillary salivary glands and paresis of the cranial nerves, especially the seventh cranial nerve (6).

Diagnosis can be achieved if histological evidence of non-necrotizing granulomas of the involved organs is obtained, in addition to typical radiological and clinical findings, and upon exclusion of other granulomatous diseases (1).

The main treatment indications are represented by the presence of dangerous clinical conditions or significant impairment in the quality of life (7). Oral corticosteroids are the first line of treatment, generally with a starting dose of 0.5–0.75 mg of prednisolone per kg of body weight daily; second-line therapy includes corticosteroid-sparing agents, such as azathioprine, methotrexate, mycophenolate mofetil, cyclosporine, cyclophosphamide, leflunomide, and hydroxychloroquine. Finally, the third-line therapy comprises anti-tumor necrosis factor-α (TNF-α) antibodies, such as infliximab or adalimumab (3).

Although sarcoidosis has been classically considered a T-cell response-mediated disease, potentially resulting from an inefficient antigen clearance, a possible pathogenetic role further played by autoimmunity in the initiation or progression of sarcoid granuloma is still unclear. The aim of this review is to deepen the immunological features of sarcoidosis, mainly focusing on the autoimmune traits of the disease, in the light of the most recent advances.

## Immunopathological considerations

### Sarcoid granuloma: Immunological analysis of a complex T-cell-mediated inflammatory response

The pathological hallmark of sarcoidosis is represented by the presence of epithelioid, non-necrotizing granulomas with lymphocytic inflammation (8). Its formation is triggered by a particular environmental or biological agent that acts in the genetically predisposed individual by activating innate immunity: in detail, macrophages and dendritic cells release pro-inflammatory cytokines, such as IL-6, IL-12, IL-18, and transforming growth factor-β (TGFβ), that modulate adaptive immune response toward a conspicuous and exaggerated activation of CD4-T lymphocytes polarized in Th1 cells and Th17.1 effector cells, with subsequent production of interferon-γ (IFNγ) and IL-17. On the other hand, cells of innate immunity determine an upregulation of the mechanistic target of the rapamycin complex 1 (mTORC1) pathway, leading to differentiation of epithelioid cells and consequent upregulation of serum amyloid A (SAA) and heat shock proteins (HSPs). Accumulation of SAA inclusion in granulomas promotes itself enhanced effector T-cell responses through innate immune receptors, such as Toll-like receptor 2 (TLR2). Impaired response of regulatory T-cells (T-reg cells) allows a persistent local T-cell activity, thus causing chronic disease.

Sarcoid granuloma can pursue two ways: a resolution one, which takes place when peptide antigens are presented by human leukocyte antigen (HLA)-DR3 molecules, expressed on dendritic cells or macrophages, and are recognized by a specific T cell receptor (TCR) β-chain variable segment 8 (TRBV8) and α-chain variable 2.3 (TRAV2.3) of a CD4-positive (TRAV2.3+TRBV22+CD4+) T cells. This particular mechanism activates an efficient immune response leading to antigen elimination and granuloma resolution. Conversely, if antigen presentation is not efficient because of the presence of other antigen-presenting cell HLA molecules or inefficient T-cell clones, a granuloma progression is established (1).

An important contribution in maintaining a pro-inflammatory state at the level of granulomas is provided by sarcoid macrophages and epithelioid cells: they are able to express and release a significative amount of CCL20, the specific ligand of the CCR6 receptor of Th17 cells. Besides, CCL20 release is stimulated by pro-inflammatory cytokines, including IL-1β and TNF-α. The interaction between CCL20 and CCR6 leads to conspicuous pulmonary recruitment of Th17 cells. In this regard, Tøndell et al. (9) demonstrated that bronchoalveolar lavage fluid (BALF) present in patients with sarcoidosis showed that the proportion of IFN-γ+ Th17 cells is greater than the controls, and also a positive correlation of IFN-γ+ Th17 cells with radiologic stage and Th1 cells. Intriguingly, the ratio between IFN-γ+ Th17 cells and T-regs expressing FoxP3 transcription factor, was markedly increased in sarcoidosis: this finding suggested a possible immune cell imbalance in sarcoidosis, especially in relation to an impaired function of regulatory T cells which have a fundamental role in maintaining immune homeostasis and preventing autoimmunity (10). T-reg cell impairment could also be deduced by analyzing the role of the inducible co-stimulator (ICOS)/ICOS-ligand axis. ICOS is a T-cell co-stimulatory molecule that promotes the proliferation and differentiation of T cells and the synthesis of IL-10, a potent anti-inflammatory cytokine (11). Sakthivel et al. (12) demonstrated that patients with sarcoidosis showed a high-level ICOS expression restricted to inflamed lung T-regs in comparison to blood T-regs of patients with sarcoidosis and to lung and blood T-regs of healthy volunteers; moreover, ICOS expression resulted significantly higher in lung T-regs than in sarcoid-specific lung effector T cells. In addition, ICOS expression was particularly elevated in the lung T-regs and in blood monocytes from patients with Löfgren's syndrome who generally present an acute onset of the disease with a frequent spontaneous resolution. For these reasons, a potential implication in ICOS/ICOS-ligand immune-regulatory axis in disease activity and resolution has been hypothesized.

A local pro-inflammatory environment is also determined by the TL1A/DR3 axis activation in the lung of patients with sarcoidosis with active disease. The interaction between TNF-like ligand 1A (TL1A) and its death domain receptor 3 (DR3) shapes a signaling pathway that determines pro-inflammatory effects, namely memory CD4+ T-cell secretion of pro-inflammatory cytokines (IFN-γ, TNF-α, IL-17), Th17 cell differentiation and proliferation, and T-reg cell proliferation with an attenuated suppressive activity (13).

The complexity of inflammatory response in sarcoidosis is attested also by the involvement of Th2- lymphocytes, which seems to occur specifically in the subset of patients with pulmonary fibrosis (PF). In fact, several evidence support the hypothesis that PF could be attributable to a switch from Th1 responses toward Th2, with a consequent release pattern of cytokines involved in PF, such as IL-13 and CCL2 (13).

### The presence of autoantibodies specific for autoimmune diseases in patients with sarcoidosis

Although cell-mediated immunity is considered the main pathogenic factor for sarcoidosis, it is found that humoral immunity may also play a certain role. This is suggested, for example, from the evidence that patients with sarcoidosis are inclined to develop autoantibodies specific for several autoimmune diseases, regardless of the presence of clinical manifestations of the respective autoimmune disease (AD). In this regard, in a recent single-center retrospective study, performed on a sample of 154 patients with sarcoidosis and 100 sex and age-matched controls, Shi et al. (14) demonstrated that patients with sarcoidosis were more prone to being autoantibody-positive compared to controls (10.4 *vs*. 3%, *p* = 0.031). In particular, in the group of patients with sarcoidosis without concomitant AD, autoantibody profiling showed the presence of anti-mitochondrial antibody-M2, anti-Ro52, anti-Ro60, anti-SSB, anti-P0, anti-CCP, anti-β2-GP, antinuclear antibodies (ANA; 1 patient presented 1:1,000 dilution titer and 1 was 1:320), anti-Sm antibody, and rheumatoid factor (RF). Interestingly, patients with sarcoidosis and autoantibodies showed higher levels of erythrocyte sedimentation rate (ESR), globulins, and CD4 cells in BALF, as well as a more advanced age compared to patients with sarcoidosis without autoantibodies. Moreover, age represented a risk factor for patients with sarcoidosis to develop autoantibodies even without the presence of AD (RR = 1.077; *p* = 0.042).

### Researching possible targets of autoimmunity in sarcoidosis

#### Evidences from proteomics

Fukushima et al. (15) identified the presence of natural autoantibodies specific for each of the chronic pulmonary diseases analyzed: chronic fibrosing idiopathic interstitial pneumonias, sarcoidosis, and autoimmune pulmonary alveolar proteinosis. The study was conducted using protein array analysis, a technique that allows the identification of novel autoantibodies related to more than 8,000 peptides and proteins that include known and candidate autoantigens. In the context of patients with a definite diagnosis of sarcoidosis, authors found the presence of sarcoidosis-specific autoantibodies against macrophage-associated antigens, including major facilitator superfamily domain containing 6 (MFSD6) and myocyte enhancer factor 2D (MEF2D); they also found autoantibodies against antigens notoriously highly expressed in the granulomatous tissue of patients with sarcoidosis, such as vonWillebrand factor (vWF) (16) and ferritin heavy chain 1 (FTH1) (17). Moreover, they found high levels of autoantibodies against annexin A11 (ANXA11), a known susceptibility gene for sarcoidosis involved in cell division, apoptosis, and neutrophil function (18) and highly expressed in immune cells such as B cells, monocytes, and myeloid cells. Due to these features, ANXA11 might have a role in granuloma formation; on the other hand, ANXA11 should not be considered a sarcoidosis-specific antigen, due to its association also with amyotrophic lateral sclerosis (19). Lastly, patients with sarcoidosis frequently showed the presence of autoantibodies against membranous and serum proteins, such as tumor necrosis factor receptor superfamily member 14 (TNFRSF14), growth differentiation factor 10 {GDF10 (BMP3)}, mucin-like protein 1 (MUCL1), ring finger and SPRY domain containing 1 (RSPRY1), RRAD and GEM like GTPase 1 (REM1), and gametocyte-specific factor 1-like (GTSF1L). The authors speculated that these autoantibodies could reflect the ongoing pathophysiology of the disease itself that could be used as biomarkers for diagnosis and prediction of disease progression.

In the same way, Häggmark et al. (20) investigated the reactivity of human IgG present in BAL and serum from patients with sarcoidosis using antigen microarrays. Their findings disclosed a high immunological reactivity, in patients with sarcoidosis, toward four proteins: ZNF688, a zinc finger protein with as-yet unclear functions in the sarcoidosis context; NCOA2, a protein that modulates nuclear hormone receptor activity; MRPL43, a mitochondrial ribosome related protein; ARFGAP1, expressed in several cell types, including alveolar macrophages that play a key role in the defense against inhaled microorganisms, also involved in sarcoidosis granuloma formation and disease outcome (21).

#### The hypothetical pathogenic role of vimentin

Vimentin is a member of type III intermediate filaments involved in several cellular functions, such as cell motility and adhesion, subcellular organization, and maintenance of cell shape (22); it has been found in Schaumann bodies (23), in asteroid bodies of sarcoidosis granulomas (24) and frequently also in fibroblasts (25).

Eberhardt et al. (26) focused their attention on the Kveim reagent, which is a treated suspension of sarcoidosis spleen tissue used for a historical *in vivo* skin diagnostic test of sarcoidosis, known as the Kveim test. This reagent, injected intradermally in a patient with suspected sarcoidosis, determines a pathognomonic reaction after 4–6 weeks, with the presence of biopsy-proven sarcoid granulomas at the injection site (27). Proteomic analysis of the Kveim reagent, compared with control spleen tissue, revealed the presence of three proteins detected only in sarcoidosis tissue: vimentin, tubulin, and α-actinin-4; the capacity of these proteins to induce a pro-inflammatory response in peripheral blood mononuclear cells (PBMCs) of patients with sarcoidosis, patients with tuberculosis, and healthy controls were tested. Among them, only vimentin stimulation induced an IFN-γ/TNF-α-based inflammatory response from sarcoidosis PBMCs; interestingly, IFN-γ and TNF-α were significantly higher in sarcoidosis-derived PBMCs than in tuberculosis-derived and healthy control-derived PBMCs.

In search of a deeper insight into the immunological role of vimentin, Kinloch et al. (28) demonstrated that B- and T-cell immunity response to vimentin in patients with sarcoidosis seems to be specifically localized in the lungs. As a first observation, the authors found that BALF titers of anti-vimentin antibodies (AVAs) were higher in patients with sarcoidosis than in healthy controls. Furthermore, while no differences were found in titers of serum AVAs between sarcoidosis and healthy controls, AVA concentrations in BALF were higher than those in matched serum samples for both sarcoidosis and controls. Moreover, results showed that BALF total autoantibodies concentrations were higher in patients with sarcoidosis than in controls and that BALF AVAs concentrations of patients with sarcoidosis were positively correlated with CD4+ T-cells clonal expansion. These findings suggest that, in sarcoidosis, vimentin could trigger an *in situ* T- and B-cell immune-mediated response.

Another hint about a possible pathogenetic role of vimentin in sarcoidosis arises from a recent study that investigated the systemic immune response to vimentin and its capacity to induce granuloma formation in a murine model of pulmonary sarcoidosis (29). Initially, the authors demonstrated that patients with sarcoidosis showed significantly (*p* = 0.0024) higher anti-vimentin IgG levels than controls and a greater prevalence of anti-vimentin IgG positivity (24/48: 50 *v*s.1/13: 7.6%, respectively; *p* = 0.0091). Then, they analyzed the effects of exposition to vimentin in pre-immunized mice to vimentin itself and in non-immunized controls mice: lung histopathology examination showed that granulomas in vimentin-immunized mice were significantly larger than those in controls (*p* < 0.0001). Interestingly, granuloma composition reflected the composition of sarcoid granuloma, including the presence of multinucleated giant cells, CD4 cells, and macrophages. Furthermore, flow cytometry analysis of BALF in vimentin-immunized mice showed higher numbers of CD45+ immune cells when compared to controls (*p* = 0.0082), most of which were represented by CD4 T cells. Finally, the analysis of vimentin-immunized mice lung RNA highlighted an upregulation, significantly higher than controls, of genes codifying for chemokines (Ccl2, Ccl3, Ccl7, Ccl8, Ccl9, Ccl12, Ccl24, Cxcl1, and Cxcl3) and chemokine receptors (Ccr2, Ccr3, Ccr4, Ccr5, and Ccr8), MHC II genes including H-2Eb1and immune-regulatory genes such as Pdcdlg2, Pdcd1, Foxp3, and Ctla4. Finally, the expression levels of Th1 and Th2 pathways genes (IFN-γ, Il-17, TNF-α, Il-6, Il-1b, Il4, Il5, Il10, Il13, Il33) were significantly higher in vimentin-immunized mice than in the control mice.

These findings also seem to suggest a probable specific pathogenetic role of vimentin in sarcoidosis disease. However, the presence of serum vimentin autoantibodies could not be considered pathognomonic of sarcoidosis disease, due to their presence in several autoimmune diseases, such as systemic erythematous lupus, antiphospholipid syndrome, and rheumatoid arthritis (30).

### Sarcoidosis and autoimmune disorders

A further tip about the possible role of autoimmunity in sarcoidosis is given from the frequent coexistence of sarcoidosis with autoimmune diseases.

In this regard, Tana et al. (31) stated that around one out of six patients with sarcoidosis could present an associated immune-mediated disease, among which autoimmune thyroid disorders, sjögren syndrome and ankylosing spondylitis are the most frequent; an association with familial Mediterranean fever, primary biliary cholangitis, hemolytic anemia, autoimmune hepatitis, antiphospholipid syndrome, immune thrombocytopenia, systemic sclerosis, psoriatic arthritis, and systemic lupus erythematosus was also described.

Regarding thyroid disorders, Nakamura et al. (32) reported a prevalence of Hashimoto's thyroiditis among patients with sarcoidosis, which is about 3–11%. In the retrospective study of Shi et al. (14), Hashimoto's thyroiditis was the most prevalent concomitant autoimmune disease among patients with sarcoidosis (3.9%). More generally, Antonelli et al. (33) reported an estimated prevalence of autoimmune thyroid disorders in patients with sarcoidosis, which were 50.7% and 22.2% among females and males, respectively.

In the field of rheumatologic diseases, Judson et al. (34) identified 15 patients with concomitant sarcoidosis and connective tissue disease, reporting a prevalence of 27% for systemic lupus erythematosus and scleroderma, 20% for rheumatoid arthritis, 13% for myositis and psoriatic arthritis. Interestingly, all patients were female and the co-occurrence between sarcoidosis and rheumatologic disease was temporally variable, in that sarcoidosis arose prior to the connective tissue disease in 53% of cases, afterward in 20%, and concomitantly in 13%. These findings showed parallelism with the authors' literature review: in a group of 53 patients with co-occurrence of sarcoidosis and connective tissue disease, most of them were female and the most prevalent rheumatologic disorders were scleroderma (46%), systemic lupus erythematosus (19%), and rheumatoid arthritis (17%).

Moreover, a coexistence of sarcoidosis and hematologic autoimmune disorders has been described, with particular reference to autoimmune hemolytic anemia and primary immune thrombocytopenia (35), the latter showing an estimated prevalence of about 2% in patients with sarcoidosis (36).

Finally, Papadopoulos et al. (37) demonstrated a higher frequency of gastric autoimmunity and gluten-associated immune reactivity in patients with sarcoidosis: in a cohort of 78 patients with sarcoidosis, H+/K+ ATPase antibodies were detected in 19 patients (24.4 *vs*. 4% in controls, *p* = 0.00015) and gliadin antibodies in 12 patients (15.4 *vs*. 8.1% in controls, *p* = 0.042).

### Evidences from the pharmacological field: The efficacy of rituximab in treating refractory sarcoidosis

Rituximab is a B-cell depleting chimeric monoclonal antibody against human CD20 (38), initially conceived for the treatment of non-Hodgkin's lymphoma (39) and successively studied for its application in several autoimmune diseases, such as rheumatoid arthritis (40), systemic lupus erythematosus (41), antiphospholipid syndrome, pemphigus, cicatricial pemphigoid, myasthenia gravis, and neuromyelitis optica (42).

Rituximab could have a useful role also in the treatment of refractory sarcoidosis: Krause et al. (43) reported a case of successful treatment of refractory cardiac sarcoidosis with rituximab while Cinetto et al. (44) described three other cases of refractory sarcoidosis with different organ involvement showing good response to rituximab treatment.

The effectiveness of rituximab in the treatment of sarcoidosis could be explained by its ability to modulate T-cellular response through interference with the role of B-lymphocytes acting as antigen-presenting cells to T-lymphocytes; on the other hand, another mechanism of action could be linked to the reduction of the levels of pathogenic or presumed pathogenic autoantibodies by indirectly reducing the amount of new plasma cells synthesis, for which B-cells are required (42). These pharmacodynamics considerations seem to suggest that also humoral immunity could have a pathogenetic influence on this disease.

### Sarcoidosis and Coronavirus Disease 2019: An interesting immunological link

Interesting immunological parallelism can be drawn between COVID-19 and sarcoidosis.

COVID-19 is an infectious disease caused by a novel coronavirus, severe acute respiratory syndrome coronavirus 2 (SARS-CoV-2), that is responsible for the ongoing pandemic state outbreaking in December 2019 in Wuhan city, the capital of Hubei province in China.

The clinical spectrum of SARS-CoV-2 infection is heterogeneous, ranging from asymptomatic infection to mild upper respiratory tract illness and severe viral pneumonia with respiratory failure and even death (45).

Zhao et al. (46) identified several common clinical and immunological features between sarcoidosis and COVID-19.

First of all, similarities between these diseases are suggested by clinical observations: several SARS-CoV-2-infected patients developed granulomatous manifestations and often a diagnosis of sarcoidosis was reached (47).

Besides, a similarity is inherent to the likely pathogenic role of angiotensin-converting enzyme (ACE) in both diseases. ACE is a component of the renin-angiotensin system (RAS) axis that is involved in the homeostasis of cardiovascular function (48) and downregulated by ACE2; serum ACE can be frequently upregulated in patients with sarcoidosis and significantly higher when compared with healthy controls so that it has been considered as a possible serum biomarker for the diagnosis of sarcoidosis or the monitoring of disease activity (49). Nonetheless, a clear relationship between sarcoidosis and ACE still has to be drawn, even though a crucial role of ACE in granuloma formation has been supposed, in light of its upregulation in activated macrophages within granulomas (47). In parallel, it has been demonstrated that SARS-CoV-2 tropism to the respiratory system is mediated by the binding of the viral particles to the ACE2 receptor on human alveolar epithelial cells, which allows the virus to enter the human body (50). Thus, the consequent reduction of ACE2 levels in the lungs may have a role in the acute pulmonary injury.

From an immunological point of view, the authors highlighted that, similarly to patients with sarcoidosis, BALF cell count analysis of hospitalized patients with COVID-19 showed accumulation of CD4+ and CD8+ T cells and CD4/CD8 ratio often higher than 1.5; blood lymphocytopenia was also detected. These findings suggest local pulmonary recruitment of lymphocytes. Interestingly, BALF cell immunophenotyping revealed high levels of Th17.1 cells, suggesting a T-cellular response polarization similar to sarcoidosis, while histopathological analysis of lung specimens from deceased patients with COVID-19 showed an interstitial and perivascular lymphocyte infiltration (51).

Moreover, an impaired function of Treg cells is also probably shared by both diseases, with a consequent role in amplifying immunoinflammatory response. COVID-19 and sarcoidosis share cytokine-releasing patterns: high levels of IL-6, IL-10, TNF-α, and IFN-γ are common in both diseases (46).

Considering the role of humoral immune response in COVID-19 and sarcoidosis, an interesting consideration is offered by the analysis of the behavior of follicular helper T cells (Tfh), a class of CD4+ T cells involved in T-cell-mediated antibody responses and in communication to B cells (52). In patients with sarcoidosis, a specific predominant subset of circulating Tfh cells seems to contribute to determining B-cell function impairment through several mechanisms: a reduction of memory B-cell subsets and a predominance of naive and activated B-cell subsets (52). In parallel, an impaired differentiation of Tfh-like cells could cause dysfunctional B-cell maturation disorders and changes in humoral immune responses in patients with COVID-19 (53). This analogy could suggest a hypothetic pathogenic role of humoral immunity in sarcoidosis.

## Discussion

Sarcoidosis is an inflammatory granulomatous disease of unclear etiology: it has been postulated that an exaggerated immunological response in the disease is triggered in genetically predisposed individuals by the exposure to hypothesized antigens: infectious agents such as *Mycobacterium tuberculosis*, atypical mycobacteria, mycoplasma species and *Propionibacterium acnes*, several fungal and viral agents; inorganic agents (aluminum, zirconium, titanium, beryllium, talc, silicates, toner dust, complex alkaline dust) and organic agents like pine tree pollen, wheat, molds, and wood dusts (13). Sarcoidosis immunological response is mainly represented by a conspicuous Th1 activation (50); however, the presence of a humoral response dysregulation is suggested by several considerations: the possible presence of autoimmune-disease-specific autoantibodies in patients with sarcoidosis, regardless of clinical evidence of the correlate autoimmune disease; the frequent co-occurrence of sarcoidosis with autoimmune diseases; the evidence of clinical and radiological response of refractory sarcoidosis to rituximab treatment.

For these reasons, in the last few years, scientific attention has been focused on the research of sarcoidosis-specific autoantibodies. In this context, proteomics highlighted the presence of autoantibodies against macrophage-associated antigens (MFSD6, MEF2D), vWF, FTH1, or against several membranous and serum proteins (15).

The main limit of these studies is linked to the essential need to demonstrate if autoantibodies detected in patients with sarcoidosis could have a significant role in the pathogenesis and in the influence of the disease course or not. In fact, the synthesis of supposed disease-specific autoantibodies in sarcoidosis could be alternatively explained in the context of the phenomenon of polyclonal hypergammaglobulinemia, classically intercepted in the sarcoidosis disease: Hunninghake et al. (54) demonstrated a markedly increased antibody production at sites of the disease activity, such as lungs and a significant positive correlation between the intensity of T-lymphocytic component of alveolitis and the numbers of bronchoalveolar cells that secrete immunoglobulins. Thus, the authors suggested that lung T-lymphocytes could be capable of direct stimulation of B-lymphocytes toward differentiation into immunoglobulin-secreting cells. Polyclonal hypergammaglobulinemia could thus represent the result of abnormal, continuous stimulation of multiple clones of B lymphocytes to produce antibodies due to an intense cellular response to putative antigens at the sites of disease activity; this kind of humoral response could lead, definitively, to the production of antibodies even against self-antigens.

The concept of continuous B-cell stimulation in sarcoidosis is also supported by case reports attesting the development of multiple myeloma generally following a sarcoidosis diagnosis: Sen et al. (55) interestingly found that patients with co-occurrence of these diseases presented a median age at the time of diagnosis of sarcoidosis, which was significantly higher than that of the general population of patients with sarcoidosis, thus suggesting a more severe and active clinical course of the disease (56): this assumption might suggest an extended half-life of B lymphocytes and plasma cells in patients with sarcoidosis, with consequent increased risk of undergoing genetic alterations leading to neoplastic transformation.

The need to demonstrate a pathogenic role of autoantibodies in sarcoidosis stems from another observation, that is the possible presence of autoimmune disease-specific autoantibodies in patients with sarcoidosis in absence of the correspondent autoimmune disease itself. This particular clinical and immunological condition seems to frame the presence of autoantibodies in sarcoidosis as the result of a non-specific humoral activation.

Another hypothesis that could explain autoantibodies synthesis in patients with sarcoidosis, in our opinion, is related to molecular mimicry, a phenomenon that occurs in the case of similarities between foreign and self-peptides, promoting activation of autoreactive T or B cells in a susceptible individual (57). Molecular mimicry has been described as the pathogenic mechanism of several autoimmune diseases, such as Guillain–Barrè syndrome (58). Similarly, an immunological model of molecular mimicry cannot be completely excluded in sarcoidosis: the exposure to a putative antigen in a genetically predisposed individual can lead on the one hand to the well-known T-cellular response; on the other hand, it could also trigger activation of autoreactive lymphocytes due to molecular similarities with self-peptides.

In parallel, the putative pathogenetic role of vimentin, a protein involved in several cellular functions, has also been investigated (26, 28, 29): despite vimentin appearing to be capable of reproducing a typical sarcoidosis inflammation in an animal model, autoantibodies against vimentin can not be considered specific of sarcoidosis disease due to their presence in several autoimmune diseases.

In contrast with these considerations about a possible non-specific role of humoral immunity in the pathogenesis of sarcoidosis, the usefulness of rituximab in treating some refractory cases offers a strong suggestion about the weight that the B-cellular activity has in the disease: drug-induced B-cells depletion is illustrative of the importance that autoimmunity covers in this immunological context.

In the light of these considerations, an interesting field of research could be related to the immunological phenotyping of patients with sarcoidosis. In other words, it could be useful to understand if patients with sarcoidosis show a different immunological setting when phenotypical features of the disease, such as organ involvement, illness duration, gender, or genetic arrangement, are considered. This could help to clarify why, notwithstanding the presence of autoimmune disease-specific autoantibodies, certain patients develop the related autoimmune disease while others do not. These observations could also have therapeutic implications: identifying factors that increase the propensity to develop humoral immunity alterations could help the physician in planning a more personalized follow-up and therapeutic strategies oriented to the control of the possible risk factors that could affect disease outcome.

Another important suggestion arises from rituximab application in sarcoidosis: in fact, it could also be interesting to identify which autoantibodies are affected by B-cell drug-induced depletion, in order to better individuate which of them has a real pathogenetic significance.

Concluding, sarcoidosis is a disease with complex immunological features, closely related to each other and influenced by environmental and genetic factors. In the pathogenesis of the disease, autoimmunity could have a certain role, as suggested by several immunological evidences, but further studies are necessarily required in order to identify immune biomarkers able to precisely assess the weight of autoimmune response in influencing disease outcome. In this regard, immunological phenotyping of patients with sarcoidosis could be useful, especially from the perspective of hoped precision medicine.

## Author contributions

LR reviewed the literature and drafted the manuscript. CS refined collected data and participated to study design. PS coordinated the study design, supervised data collection, discussed study results. All authors revised initial draft, and provided comments regarding important intellectual contribution, read, and approved the final review.

## Conflict of interest

The authors declare that the research was conducted in the absence of any commercial or financial relationships that could be construed as a potential conflict of interest.

## Publisher's note

All claims expressed in this article are solely those of the authors and do not necessarily represent those of their affiliated organizations, or those of the publisher, the editors and the reviewers. Any product that may be evaluated in this article, or claim that may be made by its manufacturer, is not guaranteed or endorsed by the publisher.
